# Pollen Morphological Characteristics of 46 Germplasm Resources of *Polygonatum* and Its Taxonomic Implications

**DOI:** 10.3390/plants13243509

**Published:** 2024-12-16

**Authors:** Mingyue Ding, Qian Xie, Lai Jiang, Lingling Liu, Wenbao Luo, Hailan Su, Qingxi Chen

**Affiliations:** 1 College of Horticulture, Fujian Agriculture and Forestry University, Fuzhou 350002, China; dingmimgy@163.com (M.D.); xieq0416@163.com (Q.X.); jianglai3605@163.com (L.J.); 14755726239@163.com (L.L.); 18159636683@163.com (W.L.); 2 Institute of Crop Sciences, Fujian Academy of Agricultural Sciences, Fuzhou 350013, China

**Keywords:** *Polygonatum*, palynology, scanning electron microscopy, cluster analysis

## Abstract

To elucidate the morphological diversity and genetic characteristics of the pollen of *Polygonatum* species, this study utilized a total of 46 samples encompassing six species and one variety of *Polygonatum*. Scanning electron microscopy (SEM) was employed to examine the morphological traits of the pollen and to analyze the evolutionary patterns and genetic relationships among *Polygonatum* species. The results indicate that the pollen grains of the 46 *Polygonatum* germplasm are uniformly characterized as monads, heteropolar, bilaterally symmetrical, atreme, and possess a mono-sulcus. They were peroblate (P/E-ratio = 0.36–0.42) in shape and medium to large (P = 18.17–27.15 μm, E = 44.11–67.07 μm) in size. And there are four types of exine ornamentation: reticulate, reticulate–perforate, reticulate–rugulate, and reticulate–verrucate. The 46 germplasm resources were classified into four clusters based on the results of a pollen morphology clustering analysis: *P. cyrtonema* and *P. macropodum* were grouped into cluster I, *P. filipes* and *P. odoratum* into cluster II, *P. kingianum* and *P. kingianum* var. *grandifolium* into cluster III, and *P. alternicirrhosum* was classified as cluster IV, standing alone. This study tentatively suggests that pollen morphology, particularly pollen size and exine ornamentation, can serve as a valuable reference for the classification, genetic relationship, and evolutionary patterns of the genus *Polygonatum.*

## 1. Introduction

*Polygonatum* is classified within the Liliaceae family according to the Engler classification system [1], however the Angiosperm Phylogeny Group (APG) reassigns it to the Asparagaceae family [2]. This genus comprises approximately 78 species globally (https://powo.science.kew.org/, accessed on 12 November 2024) and is predominantly found in the temperate regions of the Northern Hemisphere, extending primarily from the Himalayas to Japan. According to Chen and Tamura, a total of 39 species have been recorded in China, of which 20 are endemic [3]. The range of *Polygonatum* species in Fujian Province includes three recorded species: *P. cyrtonema*, *P. filipes*, and *P. odoratum*. The rhizomes of certain species in the genus *Polygonatum* are utilized as the Chinese medicinal herbs “Huangjing (Polygonati Rhizoma)” and “Yuzhu (Polygonati Odorati Rhizoma)”. [4], which hold significant medicinal, nutritional, economic, social, and cultural value [5,6]. *Polygonatum* has garnered significant attention from plant taxonomists since its inception, leading to the proposal of various taxonomic systems based on differing lines of evidence [7,8]. For instance, the genus has been classified into eight series based on morphological characteristics, including leaf arrangement and bracts [1]. Additionally, *Polygonatum* can be categorized into two groups—section *Polygonatum* and section *Verticillata*—based on chromosomal data and the micromorphological features of filaments [9]. Furthermore, an analysis of the chloroplast genome has resulted in the division of *Polygonatum* into three groups: section *Polygonatum*, section *Verticillata*, and section *Sibirica* [10,11,12,13]. Although the current classification of *Polygonatum* relies on morphological, cytological, anatomical, and molecular systematics, it remains a topic of controversy and requires further exploration through a multidisciplinary approach.

Palynology is an indispensable subject for understanding the evolution of systems and taxonomic identification. Pollen morphological characteristics are primarily governed by genetic factors and experience relatively low selective pressure, resulting in strong genetic conservation of pollen. Consequently, pollen morphology holds significant taxonomic value [14,15,16,17]. Previous studies have demonstrated that pollen morphology in *Polygonatum* varies among different species, particularly in terms of exine ornamentation, which is critical for the interspecific delimitation and identification within the genus [18,19,20,21,22]. However, the research on the pollen morphology of *Polygonatum* is limited, with most studies focusing on the species level. There remains a notable scarcity of systematic investigations into the variations in pollen within species and varieties, which are essential for determining intraspecific variation in this genus. In this study, we utilized the pollen from six species and one variety of *Polygonatum*, comprising a total of 46 germplasm resources. We employed a scanning electron microscope to observe the morphology of the pollen and to determine and analyze its morphological characteristics. The results of this study provide a new reference for the classification and phylogeny of plants within the *Polygonatum*.

## 2. Results

### 2.1. Pollen Shape and Size

**Figure 1:**
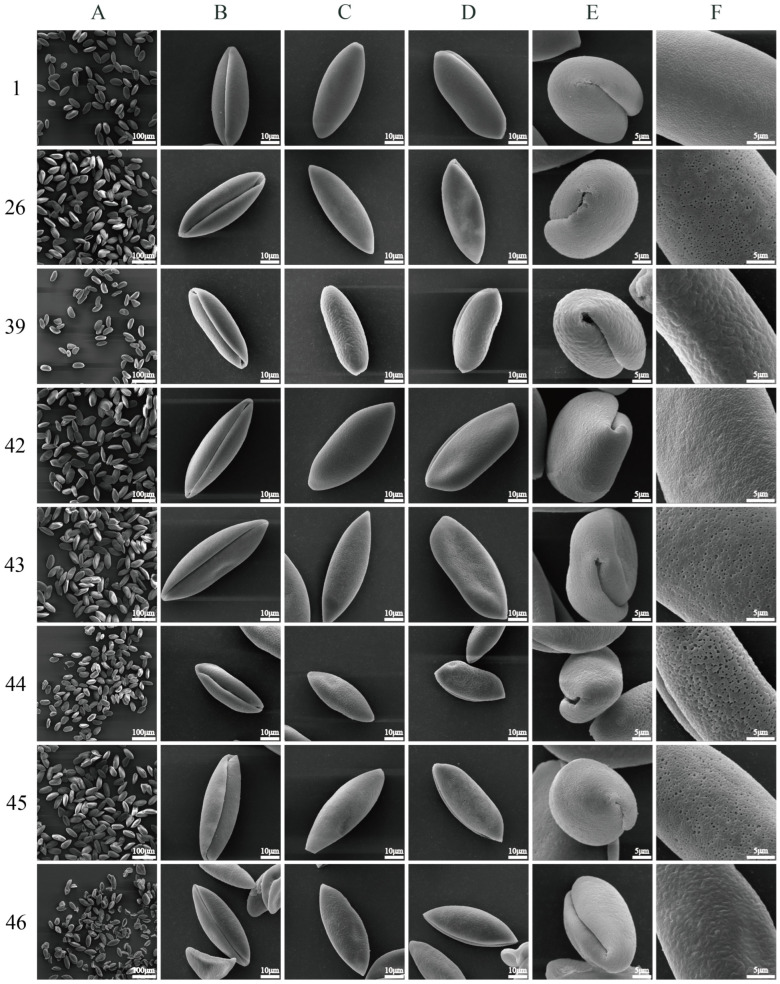
Scanning electron micrographs of pollen morphology of *Polygonatum*. 1, 26: *P. cyrtonema*. 39: *P. filipes*. 42: *P. kingianum* var. *grandifolium*. 43: *P. kingianum*. 44: *P. alternicirrhosum*. 45: *P. macropodum*. 46: *P. odoratum*. Detailed sample information is provided in Supplementary Tables S1 and S2; (**A**) pollen population (500×, bar: 100 µm); (**B**) distal view (4000×, bar: 10 µm); (**C**) proximal view (4000×, bar: 10 µm); (**D**) long equatorial view (4000×, bar: 10 µm); (**E**) short equatorial view (8000×, bar: 5 µm); (**F**) exine ornamentation (12,000×, bar: 5 µm).

As illustrated in Supplementary Table S1, Figure 1, and Supplementary Figures S1–S5, the pollen grains of 46 test materials from seven species (one variety) of *Polygonatum* are uniformly characterized as monads, heteropolar, bilaterally symmetrical, atreme, and possess a mono-sulcus. The colpus is elongated, extending straight to both ends, and is characterized by a deeper structure with a neat edge. The pollen grains of 46 test materials were classified as peroblate (P/E-ratio < 0.50). In the distal and proximal views, the pollen exhibited a long elliptical shape, while in the long equatorial view, it appeared boat shaped. In the short equatorial view, the pollen was observed to be round and kidney shaped. According to the NPC system proposed by Eltman, all 46 pollen samples were classified as type N_1_P_3_C_3_. This classification indicates that the pollen of *Polygonatum* plants exhibits a greater similarity in shape, suggesting that it can serve as a universal characterization for the pollen of this genus.

As shown in Table 1, there were highly significant differences (*p* < 0.01) in the polar axis length, equatorial axis length, P/E ratio, and the volume index of pollen among the 46 germplasm resources. According to Supplementary Table S1, the polar axis length ranges from 18.17 to 27.15 μm, with *P. kingianum* var. *grandifolium* (No. 42) exhibiting the longest polar axis and *P. alternicirrhosum* (No. 44) the shortest. The equatorial axis length varies from 44.11 to 67.07 μm, again with *P. kingianum* (No. 43) being the longest and *P. alternicirrhosum* (No. 44) the shortest. The P/E ratio ranges from 0.36 to 0.42, with *P. cyrtonema* (No. 34) showing the largest value. The volume index ranges from 28.30 to 42.61, with *P. kingianum* var. *grandifolium* (No. 42) having the largest index and *P. alternicirrhosum* (No. 44) the smallest. In summary, compared to the pollen shape, there was some differentiation in pollen size.

As shown in Table 2, when comparing the species, *P. kingianum* var. *grandifolium* exhibits the largest polar axis length, P/E ratio, and volume index among the seven species (and one variety). In contrast, *P. kingianum* has the largest equatorial axis length, while *P. alternicirrhosum* displays the smallest polar axis length, equatorial axis length, and volume index. Additionally, *P. odoratum* has the smallest P/E ratio. According to the pollen size classification, which is determined by the length of the longest axis (i.e., the length of the equatorial axis), the pollen grains of *P. alternicirrhosum* are classified as medium (25~50 μm). In contrast, the pollen grains of the remaining six species (one variety) are classified as large (50~100 μm).

### 2.2. Pollen Ornamentation

**Figure 2:**
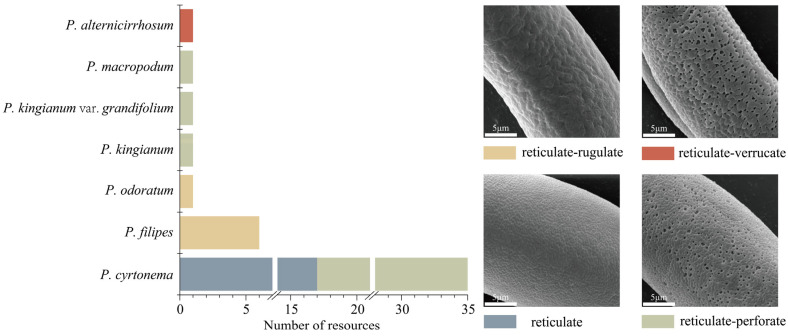
Scanning electron microphotographs of pollen ornamentation types.

As illustrated in Figure 1 and Supplementary Figures S1–S5, the exine ornamentation of the tested pollen samples can be primarily categorized into four types: reticulate, reticulate–perforate, reticulate–rugulate, and reticulate–verrucate (Figure 2). The reticulate structure is common among the 46 tested pollen samples. Reticulate ornamentation is characterized by irregularly raised lophate strips that are interconnected, forming irregular lumina. The width of the muri is inconsistent. The lumina consist of small, shallow foveolae, resulting in a slightly smooth surface. Seventeen *P. cyrtonema* germplasm resources (Figure 1 (1F), Figure S1 (3F~5F, 8F); Figure S2 (12F~13F, 15F); Figure S3 (18F, 20F, 22F, 24F~25F); Figure S4 (31F~34F)) exhibited this type of ornamentation (Figure 2).

Reticulate–perforate: The pollen surface exhibits reticulate ornamentation, while the lumina features perforations of varying sizes, predominantly concentrated in the middle of the inter-colpus area. The number and size of perforations in the pollen of different germplasm vary, allowing for a further subdivision. Specifically, 18 germplasm resources of *P. cyrtonema* (Figure 1 (26F), Figure S1 (2F, 6F~7F, 9F), Figure S2 (10F~11F, 14F, 16F~17F), Figure S3 (19F, 21F, 23F), Figure S4 (27F~30F), Figure S5 (35F)), No. 42 *P. kingianum* var. *grandifolium* (Figure 1 (42F)), No. 43 *P. kingianum* (Figure 1 (43F)), and material No. 45, *P. macropodum* (Figure 1 (45F)) are classified within this type.

Reticulate–rugulate: tThe surface of the pollen was characterized by a reticulate pattern, with irregular muri that were distinctly rugulate and curved, featuring a few perforations. This ornamentation was observed in six *P. filipes* germplasm resources (Figure 1 (39), Figure S5 (36F~38F, 40F~41F)) and in the No. 46 material of *P. odoratum* (Figure 1 (46F)).

Reticulate–verrucate: The muri become broader and thicker, resulting in flattened, verrucate, tuberous protuberances. The lumina is burrowed, relatively deep, larger, irregular, and distinctly coarser in its reticulate structure. This ornamentation was observed in the No. 44 material of *P. alternicirrhosum* (Figure 1 (44F)) and can be clearly distinguished from other species within the genus *Polygonatum*.

### 2.3. Cluster Analysis of Pollen Morphology

**Figure 3:**
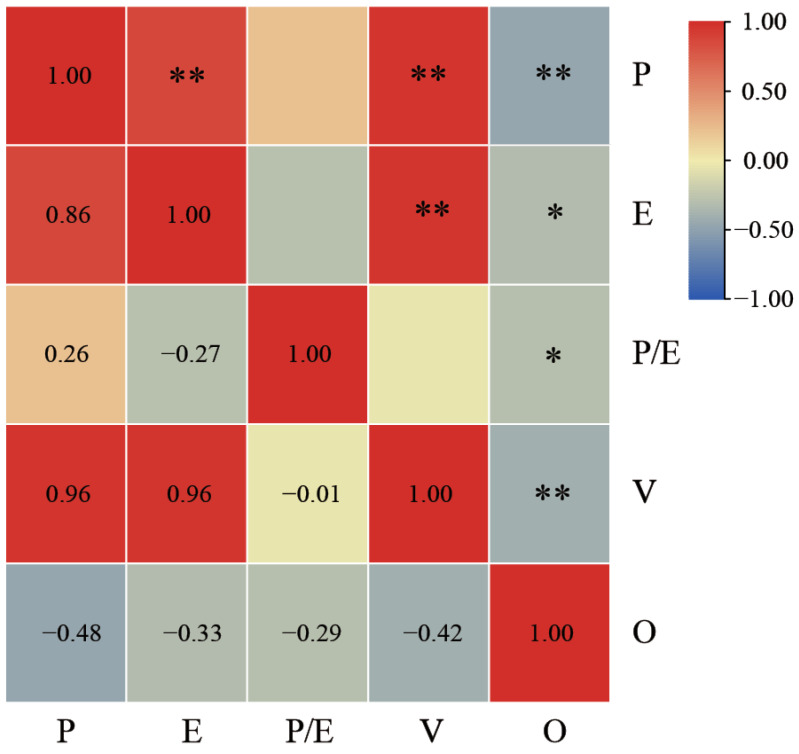
Correlation analysis of pollen morphological characteristics of *Polygonatum.* O, exine ornamentation. * Indicates a significant difference at the 0.05 level; ** indicates a significant difference at the 0.01 level.

Correlation analyses were conducted for polar axis length, equatorial axis length, P/E ratio, volume index, and exine ornamentation (Figure 3). Exine ornamentation was classified as a qualitative trait for data analysis, and values were assigned to this trait for further examination. The results indicate varying degrees of correlation among different traits. Specifically, polar axis length and equatorial axis length exhibited a highly significant positive correlation (*p* < 0.01), with a correlation coefficient of 0.86. In contrast, polar axis length demonstrated a highly significant negative correlation with exine ornamentation, yielding a correlation coefficient of −0.48. Exine ornamentation exhibited a negative correlation with other quantitative traits to varying degrees. The volume index demonstrated a positive correlation with both polar axis length and equatorial axis length, with a high correlation coefficient of 0.96. Consequently, the volume index was excluded from the subsequent Q-type cluster analysis.

**Figure 4:**
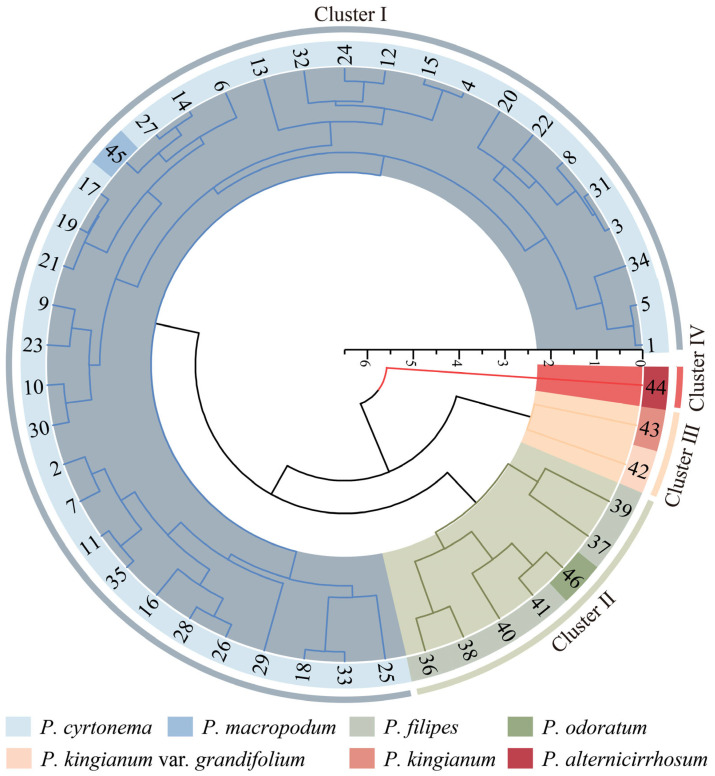
Clustering analysis of pollen morphology of 46 pollen samples from *Polygonatum*.

Based on the polar axis length, equatorial axis length, P/E ratio, and exine ornamentation, 46 test materials were subjected to Q-type cluster analysis (Figure 4). The results of the clustering indicate that these materials could be categorized into four distinct clusters. Cluster I comprises 36 germplasm resources. This cluster includes 35 resources of *P. cyrtonema* and material No. 45 of *P. macropodum*, indicating a closer genetic relationship between these two species. Additionally, exine ornamentation observed exhibits both reticulate and reticulate–perforate patterns. Cluster II comprises seven germplasm resources. This cluster includes six resources of *P. filipes* and material No. 46 of *P. odoratum*, with exine ornamentation exhibiting a reticulate–rugulate pattern. Cluster III contains only two germplasm resources: No. 43 of *P. kingianum* and No. 42 of *P. kingianum* var. *grandifolium*. The characteristics of these species include longer pollen polar and equatorial axis lengths, a larger volume index, larger pollen grains, and exine ornamentation that is reticulate–perforate. Cluster IV contained only material No. 44 of *P. alternicirrhosum*, whose pollen was the smallest of the 46 test materials and whose exine ornamentation was reticulate–verrucate. Therefore, according to the size and exine ornamentation of pollen, these characteristics reflect the genetic relationship among various *Polygonatum* germplasm resources to a certain extent, which holds significant taxonomic value. Furthermore, Figure 4 illustrates that there is no apparent correlation between the genetic distance and the geographic distance among the tested materials.

## 3. Discussion

### 3.1. Pollen Morphological Characteristics

Pollen serves as the reproductive organ of seed plants, carrying a substantial amount of genetic information necessary for the reproduction of offspring. The high degree of genetic conservatism in its structure renders pollen traits a valuable tool for studying plant origins, phylogeny, and genetic relationship [23,24]. In the present study, we show that the pollen of the genus *Polygonatum* exhibits certain commonalities, the pollen grains were bilaterally symmetrical heteropolar monads with a mono-sulcus, which is in agreement with the results of the existing studies [18,20,25].

In this study, we observed that the pollen shapes of 46 *Polygonatum* germplasm resources were relatively uniform, all exhibiting a peroblate shape. However, there were highly significant differences in pollen size-related indices (Table 1). This suggests that pollen shape is more genetically conserved, a conclusion supported by findings in other species indicating [26,27,28] that pollen shape exhibits greater genetic stability and less variability compared to pollen size. However, pollen size does demonstrate a certain degree of differentiation. This observation aligns with the findings of He et al. [29] regarding kiwifruit pollen. The polar axis length, equatorial axis length, and P/E ratio reported by Deng et al. [20], Wang et al. [19], and Zheng et al. [22] differ from those observed in the present study, likely due to variations in the sample collection and processing methods. Specifically, Deng et al. [20] and Wang et al. [19] utilized herbarium specimens, while Deng et al. [20] and Zheng et al. [22] employed the acetolysis method for pollen treatment prior to measuring pollen size under a light microscope. This process may have subjected the pollen to various factors, including heating and the dehydrating effects of the sulfuric acid solution, which could lead to deformation and measurement bias. The existing research supports the notion that different preparation methods can significantly influence pollen size [30,31,32,33].

In terms of exine ornamentation, the test materials exhibited four distinct types of ornamentation, with notable differences observed between species and germplasm. Among these, 35 germplasm resources of *P. cyrtonema* displayed two types of ornamentation: reticulate and reticulate–perforate. This finding aligns with the observations of Ali et al. [18], which indicated that *P. cyrtonema* germplasm demonstrates some degree of differentiation. Moreover, exine ornamentation reflects varying degrees of variation and genetic diversity. Deng et al. [20] characterized the exine ornamentation of the pollen of *P. cyrtonema* and *P. filipes* as verrucate and perforate, respectively. In contrast, Wang et al. [19] described the ornamentation of *P. kingianum* and *P. odoratum* as perforate and rugulate–perforate, respectively. These descriptions differ significantly from the findings of the present study. However, upon comparing the figures from the current study with those of previous authors, consistency was observed, suggesting that the variations in the classification of pollen ornamentation may stem from changes in the terminology. The pollen morphology of *P. kingianum* var. *grandifolium* and *P. alternicirrhosum* is reported for the first time, contributing new data to the study of palynology within the genus *Polygonatum*.

### 3.2. Evolutionary Trends in Pollen Morphological Characteristicss

The morphological features of pollen, particularly exine ornamentation and aperture characteristics, are crucial for elucidating the phylogeny, genetic relationship, and systematic classification of *Polygonatum*. Pollen morphological characteristics are the result of gene expression and have strong genetic stability, so pollen can reflect the general law of plant evolution [34]. The research indicates that pollen size can serve as an indicator of angiosperm evolution. It is posited that more primitive angiosperms tend to produce relatively large pollen grains, whereas a decrease in pollen volume is associated with a higher evolutionary level [35]. In this study, 46 germplasm resources were examined, of which 97.83% exhibited large pollen grains. Notably, only material No. 44, *P. alternicirrhosum*, displayed medium pollen grains. This observation suggests that *Polygonatum* plants are largely in a primitive stage of evolution, whereas *P. alternicirrhosum* (material No. 44) appears to be relatively more evolved. Its pollen size is transitioning from large to medium, a finding that aligns with the results reported by Deng et al. [20].

The aperture (colpus), which is controlled by strict genetic factors, is one of the important features of pollen’s morphology, and thus it reflects the evolutionary trend of pollen’s morphology [27]. Wang et al. [34] proposed a general evolutionary sequence for apertures (colpus): unfixed apertures (colpus) → proximal apertures (colpus) → distal apertures (colpus) → equatorial apertures (colpus). All pollen examined in this study exhibited a mono-sulcus and conformed to the N_1_P_3_C_3_ type, further suggesting that *Polygonatum* represents a more primitive form of angiosperm.

It is now widely accepted that the exine ornamentation of angiosperm pollen evolved from an unstructured, non-perforated layer to a tectum with perforations. Based on this evolution, two distinct trends have emerged: (1) expansion through perforation leading to the formation of a half tectum → no perforation → lack of tectum; (2) degrading through perforation to a secondary tectum with no perforation → secondary granules–columnar trace → secondary unstructured layer [34]. Deng et al. [20] also proposed a similar speculation. Based on the pollen size, material No. 44, *P. alternicirrhosum*, is the most evolved among the 46 test materials. Its lumina ornamentation is characterized by relatively deeper and larger reticulate–verrucate patterns. The lumina of the remaining 45 germplasm resources were small, suggesting that the evolution of *Polygonatum* pollen may have occurred through the continuous expansion of perforation. However, since this study examined only seven species (one variety) of *Polygonatum*, there are limitations to the findings, and accurate conclusions must be drawn from further in-depth studies of their phylogeny.

### 3.3. Genetic Relationship Analysis Based on Pollen Morphological Characteristicss

Pollen size and exine ornamentation can to some extent provide a reference point for plant classification and genetic relationship [36,37]. According to the clustering results of the four indicators related to pollen morphology (Figure 4), when the Euclidean genetic distance was set at 3, *P. alternicirrhosum* was classified into a distinct group. Meanwhile, *P. kingianum* and *P. kingianum* var. *grandifolium* were grouped together. Additionally, *P. cyrtonema* and *P. macropodum* were classified into cluster I. *P. filipes* and *P. odoratum* were categorized into cluster II, indicating a closer genetic relationship. These findings suggest that pollen morphological characteristics can reflect the differences among species of *Polygonatum* to a certain extent. When the Euclidean genetic distance was 3.5, *P. cyrtonema*, *P. macropodum*, *P. filipes*, and *P. odoratum* were classified into one group, while *P. alternicirrhosum* remained clustered in a separate group. Additionally, *P. kingianum* and *P. kingianum* var. *grandifolium* were grouped together. This classification aligns with Tang’s systematic categorization based on morphological traits, including leaf arrangement and bracts. It categorizes *P. cyrtonema*, *P. macropodum*, *P. filipes*, and *P. odoratum* within the series *Alternifolia* Baker, while *P. kingianum* is placed in the series *Kingiana*, and *P. alternicirrhosum* is classified under the series *Verticillata* [1]. The results of the cluster analysis align with the morphological characteristics of the subjects, a division that is further corroborated at the molecular level by the findings of Li et al. [38] and Zhu et al. [39]. As illustrated in Figure 4, crossover was observed not only between species but also geographic origin. For instance, the germplasm resources of *P. cyrtonema* from Fujian Province, China, did not cluster together; instead, they were cross-clustered with those from other provinces. This observation indicates that there is no significant correlation between genetic distances and the geographic origin of the germplasm.

## 4. Materials and Methods

### 4.1. Plant Material

The test materials comprised six species and one variety, totaling 46 germplasm resources collected from ten provinces in China: Fujian, Hunan, Hubei, Anhui, Sichuan, Guangxi, Jiangxi, Zhejiang, Guangdong, and Shandong, during the period from April to May 2023. More than 30 plants were collected from each germplasm species and planted in the Huangjing Germplasm Resource Nursery of Fujian Agriculture and Forestry University (26°5′16″ N, 119°13′40″ E; 108 m above sea level) for uniform cultivation and management. All germplasm experienced aboveground stem dieback from October to December 2023, but re-emerged in March 2024, with mature, fresh pollen collected following flowering in April. Detailed information regarding the 46 germplasm resources is available in Supplementary Table S2.

### 4.2. Experimental Method

#### 4.2.1. Pollen Collection, Preservation, and Treatment

Fresh flowers in bud were randomly collected at approximately 10:00 a.m. on a sunny day. The anthers were dried using the room-temperature silica gel drying method. Using pointed tweezers, the anthers were carefully removed and placed into a sulfate paper bag, which was then sealed in a box containing color-changing silica gel at room temperature for a duration of 48 to 72 h [40]. Once the pollen was dispersed, and the flowers were sealed in a drying tube and stored in a refrigerator at −20 °C, ensuring protection from light for future use.

#### 4.2.2. Scanning Electron Microscopy Observation

Pollen observation refers to the method of Li et al. [15] with slight modifications. The dried pollen was evenly spread on the sample stage, which was adhered with conductive adhesive, using a toothpick. Excess pollen was removed with an ear wash ball, and the sample was then gold-coated using an ion sputterer (GVC-1000, KYKY, Beijing, China) for 60 s. Subsequently, the sample was placed in a benchtop scanning electron microscope (ZEM15C, Zeptools, Tongling, China) for observation and imaging [15]. The entire pollen structure, including distal, proximal, and exine ornamentations, was examined at a voltage of 15 kV. Additionally, long equatorial and short equatorial features were also observed at the same voltage, resulting in clear photographs.

### 4.3. Pollen Morphology Description and Data Statistics

Twenty pollen grains from each germplasm resource were randomly selected, and the polar axis length (P), equatorial axis length (E), the ratio of polar axis length to equatorial axis length (P/E), and the volume index (V) of the pollen grains were determined using ImageJ 1.8.0 software. The volume index was calculated as V = $\sqrt{P\times E}$. The descriptive terms for pollen polarity, symmetry, shape, size, apertures, exine ornamentation, and the pollen classification system (NPC system) were derived from the works of Li et al. [41], Wang et al. [34], and Erdtman [42].

Descriptive statistics for quantitative traits related to pollen morphology were observed using IBM SPSS Statistics 26 software. The results are presented as mean ± standard deviation and were subjected to one-way analysis of variance (ANOVA). The data were Z-score normalized and subsequently clustered using Origin 2021, employing the average clustering method and Euclidean cluster type. Pearson correlation served as the metric, and TB-tools was utilized for plotting.

Pollen shape categorization based on the P/E ratio was as follows: a P/E ratio greater than 2 indicates a perprolate shape; a ratio between 1.33 and 2 signifies a prolate shape; a ratio between 1.14 and 1.33 corresponds to a subprolate shape; a ratio between 0.88 and 1.14 is classified as spheroidal; a ratio between 0.50 and 0.88 indicates an oblate shape; and a ratio less than 0.50 is considered peroblate [42].

Pollen size, measured as the length of the longest axis, can be categorized as follows: very small pollen if the longest axis is less than 10 μm; small pollen if it measures between 10 and 25 μm; medium pollen if it ranges from 25 to 50 μm; large pollen if it is between 50 and 100 μm; very large pollen if it spans from 100 to 200 μm; and huge pollen if the longest axis exceeds 200 μm [42].

The NPC system categorizes apertures based on three criteria: N indicates the number of apertures, with classifications including atreme (N_0_), nomotreme (where the number can range from 1 in N_1_ to 6 in N_6_, or exceed 6 in N_7_), and anomotreme (N_8_). P denotes the location of the apertures, where P_0_ signifies an unclear location, P_1_ indicates a catatreme, P_2_ represents one catatreme and one anatreme, P_3_ refers to an anatreme, P_4_ denotes a zonotreme, P_5_ indicates two or more zonotreme, and P_6_ represents a pantotreme. Lastly, C describes the character or shape of the apertures; C_0_ indicates that the character cannot be determined, C_1_ denotes a leptoma, C_2_ refers to a trichotomocolpate, C_3_ indicates colpus, C_4_ represents a porate, C_5_ denotes a colp-orate, and C6 indicates a pororate [42].

## 5. Conclusions

The 46 *Polygonatum* germplasm resources comprised six species and one variety: *P. cyrtonema*, *P. filipes*, *P. kingianum*, *P. kingianum* var. *grandifolium*, *P. alternicirrhosum*, *P. macropodum*, and *P. odoratum*. The pollen grains are characterized as monads, heteropolar, bilaterally symmetrical, atreme, and possess a mono-sulcus. The pollen is characterized by a peroblate shape (P/E ratio = 0.36–0.42) and falls within the medium to large size grade (P = 18.17–27.15 μm, E = 44.11–67.07 μm). The pollen size is undergoing an evolutionary transition from large to medium. There were four types of exine ornamentation observed: reticulate and reticulate–perforate in *P. cyrtonema*; reticulate–rugulate in *P. filipes* and *P. odoratum*; and reticulate–perforate in *P. kingianum*, *P. kingianum* var. *grandifolium*, and *P. macropodum*. Additionally, *P. alternicirrhosum* exhibited an reticulate–verrucate exine ornamentation. The clustering results based on pollen morphological characteristics provide substantial support for the morphological classification treatment. Furthermore, the clustering analysis revealed no significant correlation between genetic distance and geographic distance among the test materials. These findings offer a valuable reference for the classification of *Polygonatum*.

## Figures and Tables

**Table 1:** Analysis of variance of quantitative traits of 46 pollen samples from *Polygonatum*.

Trait	*SS*	df	*MS*	*F*	*p*
P/μm	2163.229	45	48.072	26.248	0.000
E/μm	14,410.124	45	320.225	45.955	0.000
P/E	0.193	45	0.004	7.519	0.000
V	5189.680	45	115.326	45.347	0.000

Notes: P, polar axis length; E, equatorial axis length; P/E, ratio of polar axis length to equatorial axis length; V, volume index.

**Table 2:** Comparison of pollen morphological characters among species of *Polygonatum*.

Species	P/μm	E/μm	P/E	V
*P. cyrtonema*	22.52 ± 0.79	58.11 ± 2.63	0.39 ± 0.01	36.15 ± 1.31
*P. filipes*	20.18 ± 1.25	53.75 ± 3.35	0.38 ± 0.02	32.90 ± 1.91
*P. kingianum* var. *grandifolium*	27.15 ± 1.18	66.92 ± 2.84	0.41 ± 0.02	42.61 ± 1.46
*P. kingianum*	25.32 ± 1.49	67.07 ± 2.44	0.38 ± 0.02	41.20 ± 1.72
*P. alternicirrhosum*	18.17 ± 1.36	44.11 ± 2.33	0.41 ± 0.03	28.30 ± 1.55
*P. macropodum*	22.23 ± 1.42	57.14 ± 2.81	0.39 ± 0.03	35.62 ± 1.66
*P. odoratum*	20.42 ± 1.16	54.55 ± 2.52	0.37 ± 0.02	33.36 ± 1.43

## Data Availability

All relevant data can be found within the manuscript and its Supplementary Materials. Further inquiries can be directed to the corresponding authors.

## Supplementary Materials

The following supporting information can be downloaded at: https://www.mdpi.com/article/10.3390/plants13243509/s1, Figure S1: Scanning electron micrographs of pollen morphology of *Polygonatum*. 2–9: *P. cyrtonema*; Figure S2: Scanning electron micrographs of pollen morphology of *Polygonatum*. 10–17: *P. cyrtonema*; Figure S3: Scanning electron micrographs of pollen morphology of *Polygonatum*. 18–25: *P. cyrtonema*; Figure S4: Scanning electron micrographs of pollen morphology of *Polygonatum*. 27–34: *P. cyrtonema*; Figure S5: Scanning electron micrographs of pollen morphology of *Polygonatum*. 35: *P. cyrtonema*. 36–38, 40–41: *P. filipes*; Table S1: Morphological characteristics and trait statistics of 46 pollen samples from *Polygonatum*; Table S2: Information on the material of *Polygonatum* for testing.
