# Supplementary material for: Pollen Morphological Characteristics of 46 Germplasm Resources of Polygonatum and Its Taxonomic Implications
Source: Plants (Basel). 2024 Dec 16;13(24):3509. doi: 10.3390/plants13243509 (PMC11677909; doi:10.3390/plants13243509)
Supplement: Supplementary file 1 [file plants-13-03509-s001.zip › plants-3276996-supplementary.pdf]

Table S1. Morphological characteristics and trait statistics of 46 pollen samples from *Polygonatum.*

Number	P/μm	E/μm	P/E	V	O
1	22.76±1.17	55.46±2.36	0.41±0.02	35.52±1.40	reticulate
2	23.25±1.30	61.13±2.29	0.38±0.02	37.68±1.40	reticulate-perforate
3	23.30±1.32	58.30±1.92	0.40±0.03	36.84±1.28	reticulate
4	21.37±1.29	56.78±3.49	0.38±0.03	34.81±1.76	reticulate
5	22.56±1.84	55.68±3.45	0.41±0.03	35.42±2.28	reticulate
6	22.75±1.40	57.80±2.51	0.39±0.03	36.24±1.29	reticulate-perforate
7	22.82±1.77	59.48±2.21	0.38±0.03	36.81±1.75	reticulate-perforate
8	23.47±1.07	58.49±2.13	0.40±0.02	37.05±1.27	reticulate
9	23.01±2.17	57.17±3.23	0.40±0.03	36.25±2.47	reticulate-perforate
10	22.30±1.45	56.14±1.92	0.40±0.02	35.37±1.57	reticulate-perforate
11	22.52±1.71	60.70±4.16	0.37±0.03	36.95±2.25	reticulate-perforate
12	21.97±1.80	56.74±2.67	0.39±0.03	35.29±2.08	reticulate
13	21.58±1.16	53.45±1.79	0.40±0.02	33.95±1.18	reticulate
14	21.81±1.48	56.14±3.44	0.39±0.03	34.96±1.72	reticulate-perforate
15	21.28±1.27	56.46±2.84	0.38±0.02	34.65±1.74	reticulate
16	23.92±1.58	63.06±2.92	0.38±0.02	38.82±1.92	reticulate-perforate
17	21.84±1.31	57.08±3.21	0.38±0.03	35.29±1.71	reticulate-perforate
18	22.03±1.43	59.00±2.57	0.37±0.02	36.04±1.73	reticulate
19	21.81±1.13	57.84±2.44	0.38±0.02	35.51±1.42	reticulate-perforate
20	22.88±1.75	59.00±2.41	0.39±0.03	36.71±1.77	reticulate
21	22.12±1.42	57.58±2.99	0.38±0.02	35.67±1.82	reticulate-perforate
22	23.6±1.15	57.94±2.92	0.41±0.01	36.97±1.71	reticulate
23	23.48±1.32	58.74±2.21	0.40±0.02	37.13±1.46	reticulate-perforate
24	21.89±1.01	55.69±1.48	0.39±0.02	34.90±1.03	reticulate
25	23.61±0.74	62.54±2.34	0.38±0.02	38.42±1.07	reticulate
26	23.35±1.32	64.07±2.20	0.37±0.03	38.65±0.99	reticulate-perforate
27	21.88±0.89	56.51±2.18	0.39±0.02	35.15±1.10	reticulate-perforate

28	23.79±1.27	63.77±3.53	0.37±0.02	38.94±1.82	reticulate-perforate
29	21.62±1.37	59.45±2.57	0.36±0.02	35.84±1.60	reticulate-perforate
30	21.91±1.07	55.09±1.83	0.40±0.02	34.74±1.22	reticulate-perforate
31	23.21±1.29	57.94±2.05	0.40±0.02	36.65±1.37	reticulate
32	21.05±1.17	54.52±2.56	0.39±0.02	33.86±1.41	reticulate
33	22.15±1.25	59.78±2.58	0.37±0.02	36.38±1.44	reticulate
34	22.79±1.26	54.38±2.92	0.42±0.02	35.19±1.71	reticulate
35	22.41±1.25	59.96±2.93	0.37±0.02	36.65±1.69	reticulate-perforate
36	18.61±1.02	50.81±2.13	0.37±0.02	30.74±1.37	reticulate-rugulate
37	21.47±1.40	54.59±2.98	0.39±0.03	34.22±1.66	reticulate-rugulate
38	18.77±1.25	52.11±1.54	0.36±0.03	31.25±1.13	reticulate-rugulate
39	20.04±1.31	50.11±2.38	0.40±0.03	31.67±1.38	reticulate-rugulate
40	21.27±0.77	58.78±2.61	0.36±0.03	35.33±0.67	reticulate-rugulate
41	20.89±1.19	56.09±2.84	0.37±0.02	34.21±1.57	reticulate-rugulate
42	27.15±1.18	66.92±2.84	0.41±0.02	42.61±1.46	reticulate-perforate
43	25.32±1.49	67.07±2.44	0.38±0.02	41.20±1.72	reticulate-perforate
44	18.17±1.36	44.11±2.33	0.41±0.03	28.30±1.55	reticulate-verrucate
45	22.23±1.42	57.14±2.81	0.39±0.03	35.62±1.66	reticulate-perforate
46	20.42±1.16	54.55±2.52	0.37±0.02	33.36±1.43	reticulate-rugulate

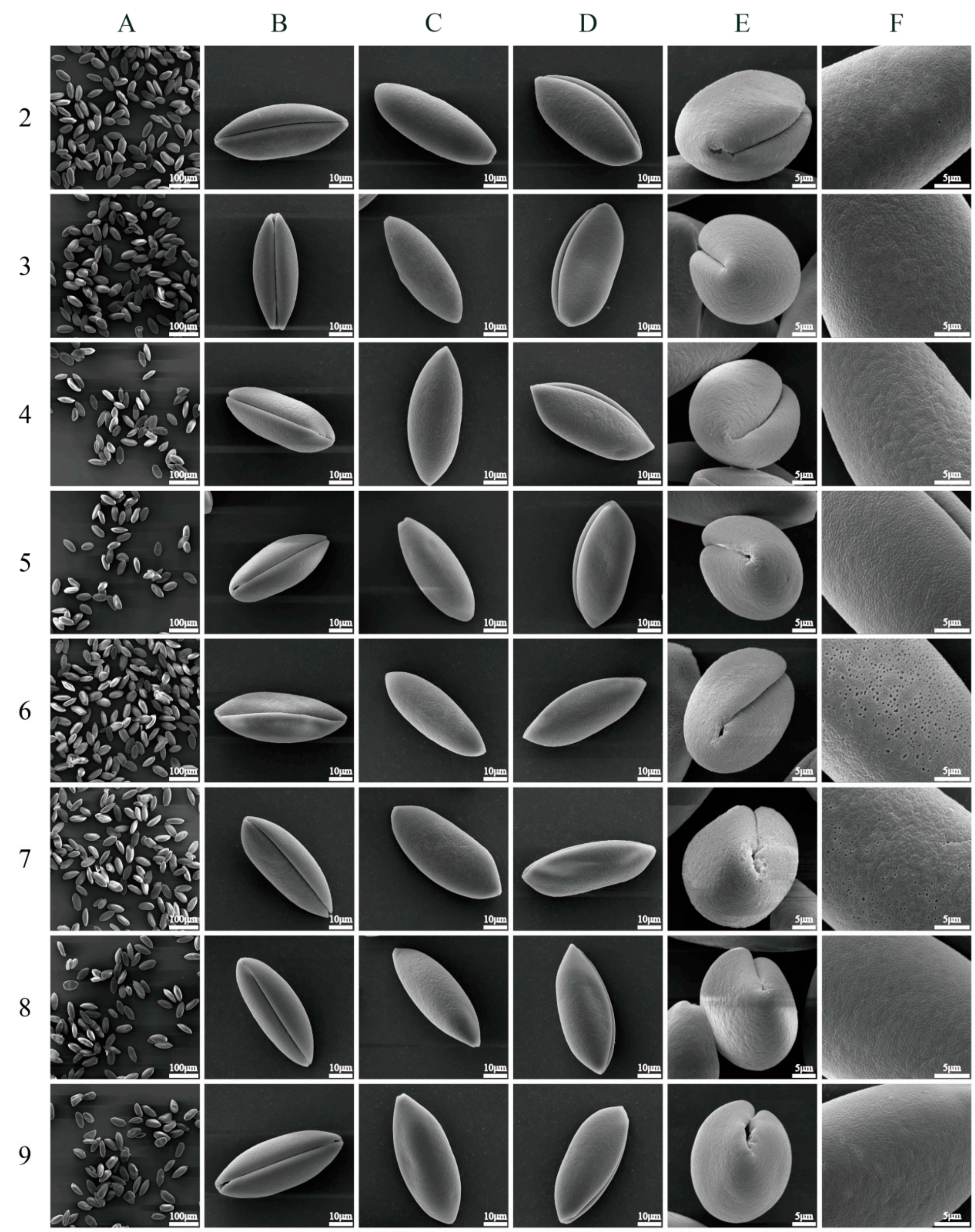

Figure S1. Scanning electron micrographs of pollen morphology of *Polygonatum.* 2-9: *P. cyrtonema.* Detailed samples information is provided in Supplementary Table S1 and Table S2; A. pollen population (500×, bar: 100 μm); B. distal view (4000×, Bar: 10 μm); C: proximal view (4000×, bar: 10 μm); D. long equatorial view (4000×, bar: 10 μm); E. short equatorial view (8000×, bar: 5 μm); F. exine ornamentation (12000×, bar: 5 μm), the same below.

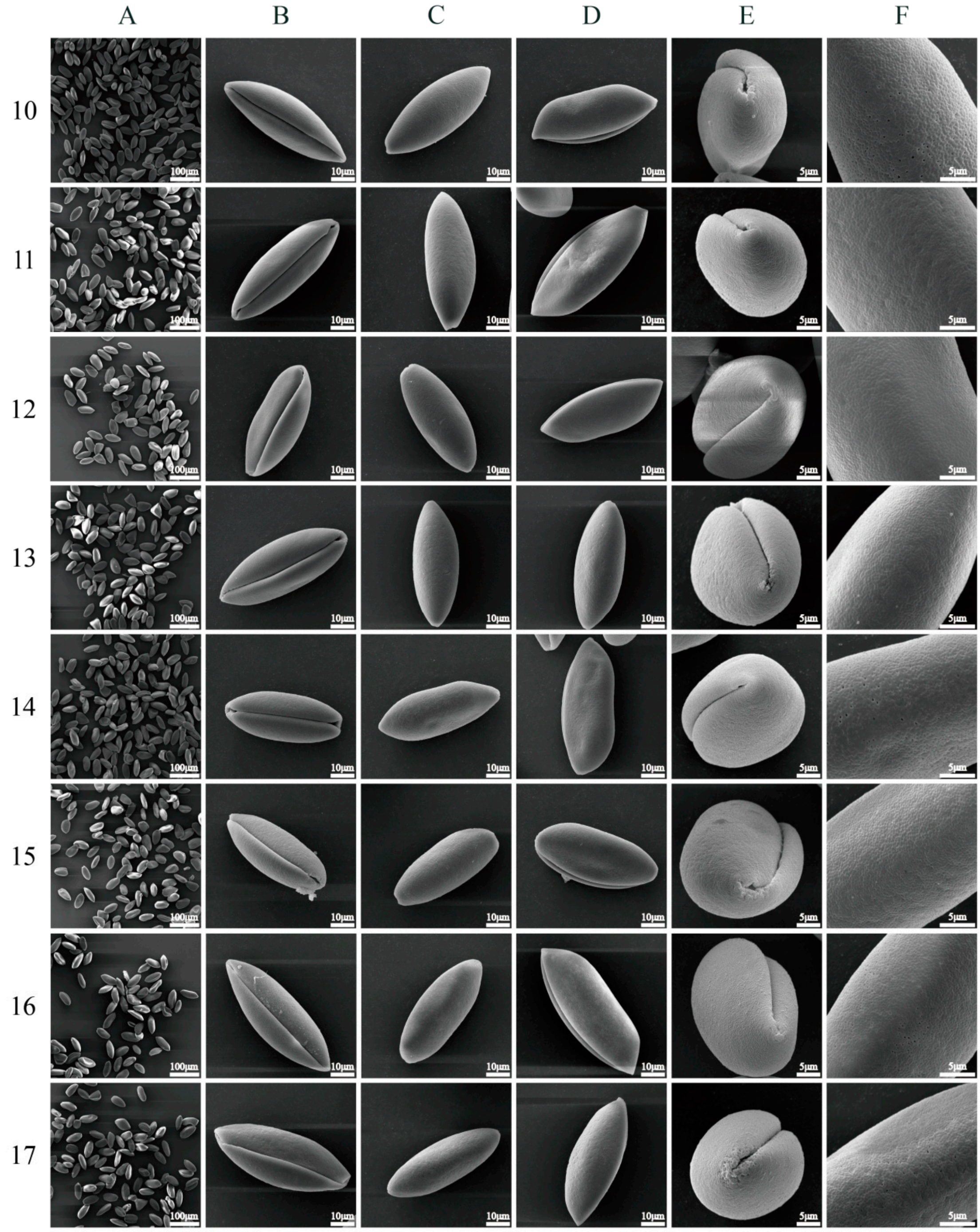

Figure S2. Scanning electron micrographs of pollen morphology of *Polygonatum.* 10-17: *P. cyrtonema.*

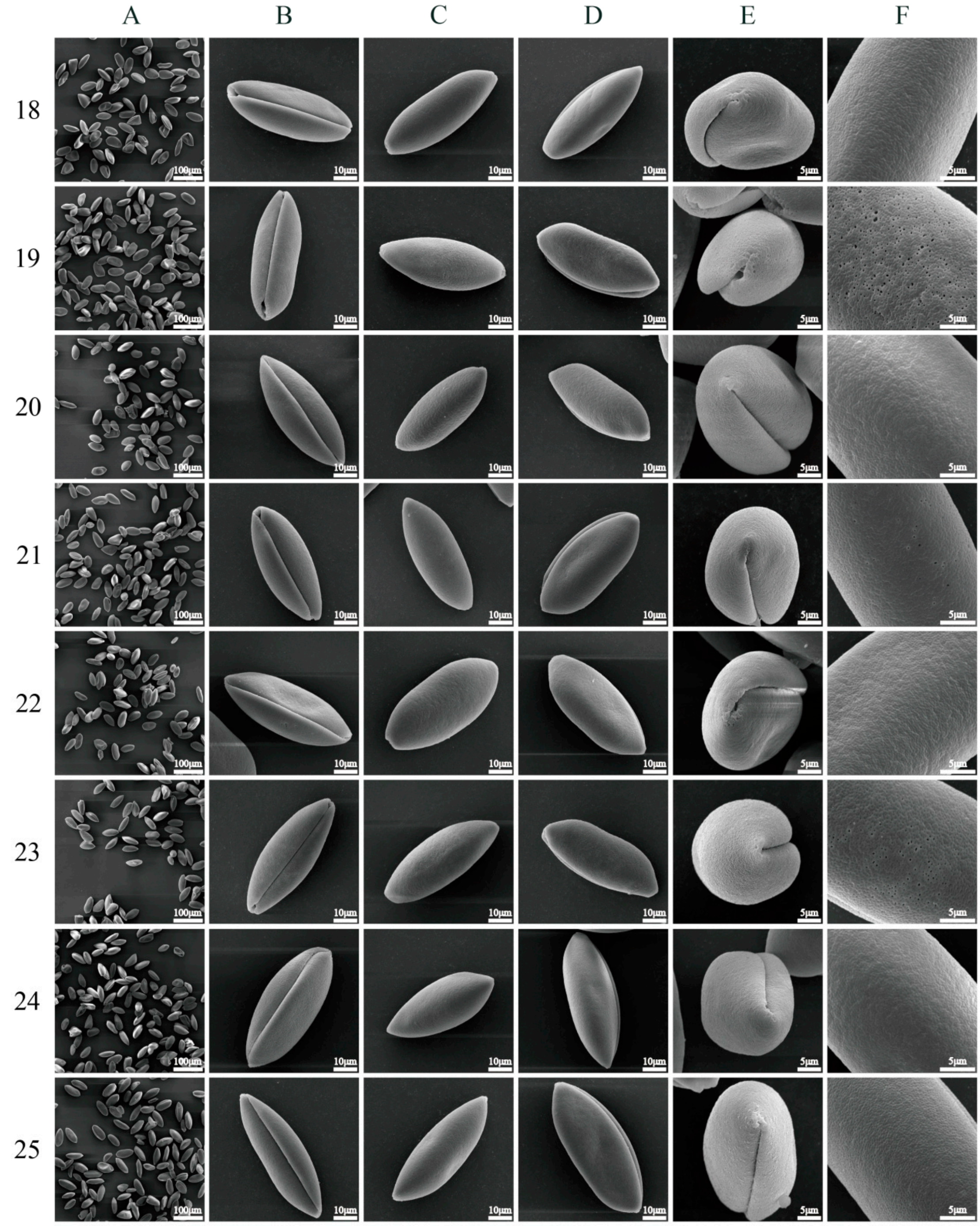

Figure S3. Scanning electron micrographs of pollen morphology of *Polygonatum.* 18-25: *P. cyrtonema.*

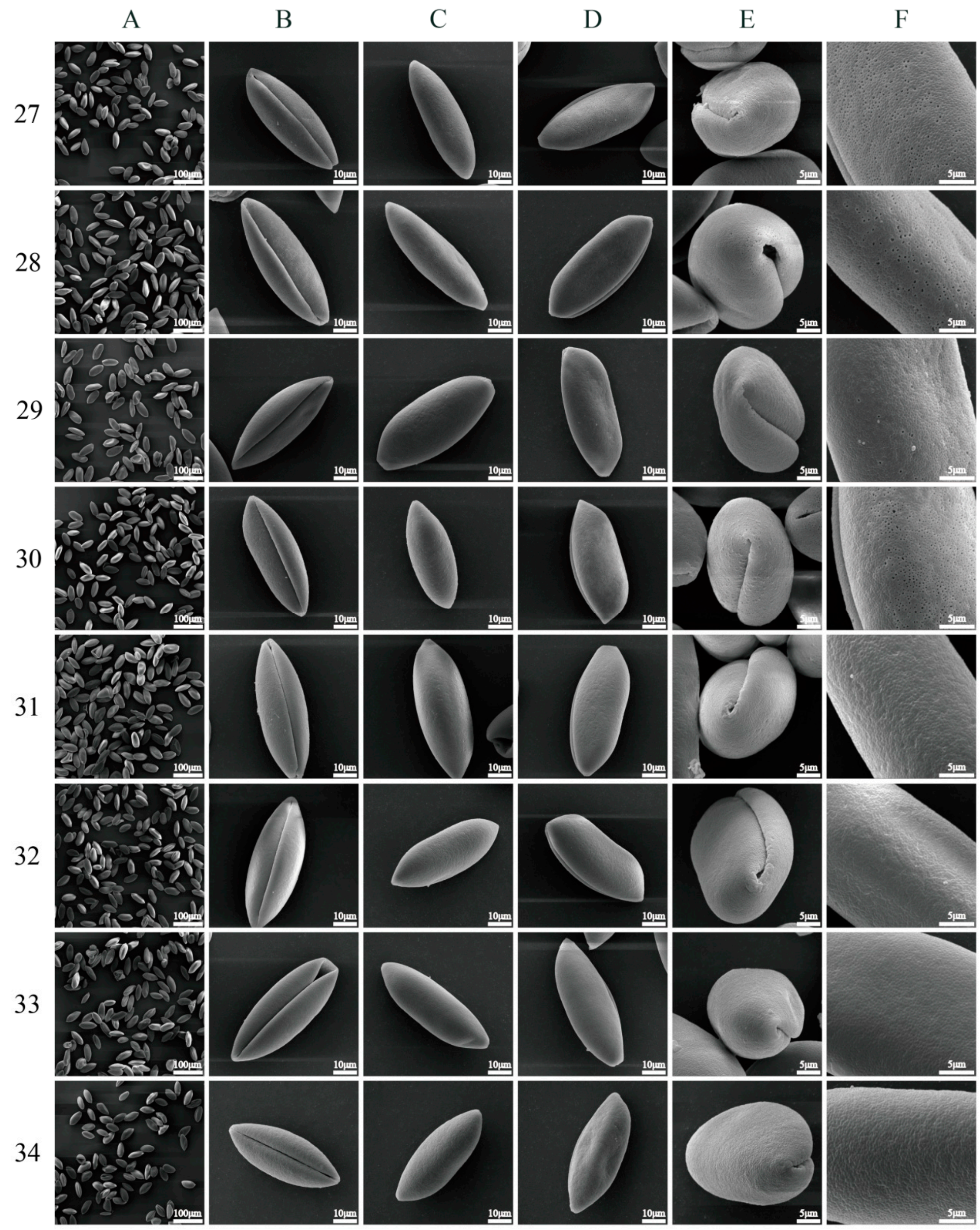

Figure S4. Scanning electron micrographs of pollen morphology of *Polygonatum*. 27-34: *P. cyrtonema*.

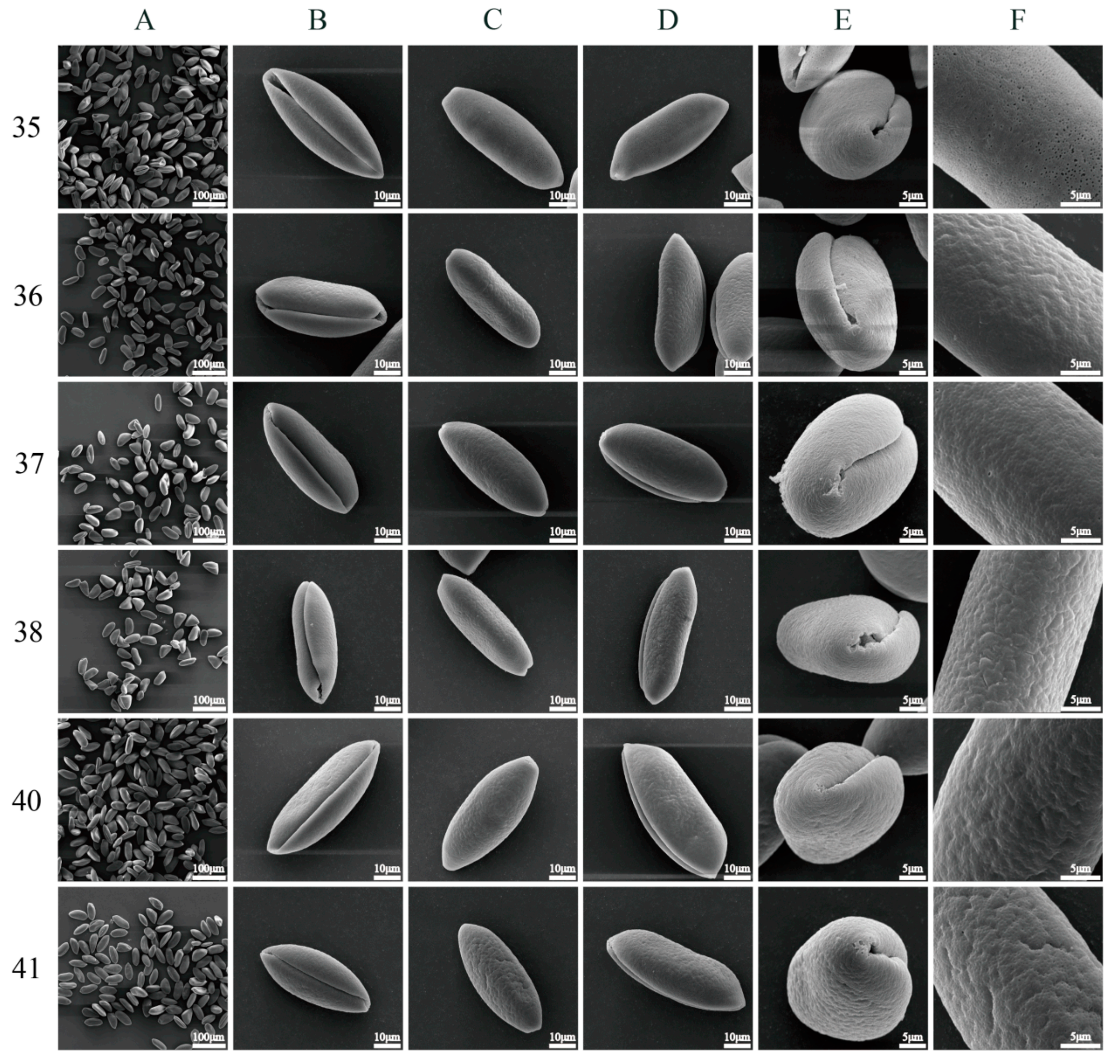

Figure S5. Scanning electron micrographs of pollen morphology of *Polygonatum*. 35: *P. cyrtonema*. 36-38, 40-41: *P. filipes.*

Table S2. Information on the material of *Polygonatum* for testing.

Number	Species	Locality	Number	Species	Locality
1	*P. cyrtonema*	Guangze, Fujian, China	24	*P. cyrtonema*	Chizhou, Anhui, China
2	*P. cyrtonema*	Shaoyang, Hunan, China	25	*P. cyrtonema*	Hezhou, Guangxi, China
3	*P. cyrtonema*	Shaowu, Fujian, China	26	*P. cyrtonema*	Hezhou, Guangxi, China
4	*P. cyrtonema*	Shaowu, Fujian, China	27	*P. cyrtonema*	Huanggang, Hubei, China
5	*P. cyrtonema*	Shaowu, Fujian, China	28	*P. cyrtonema*	Huaihua, Hunan, China
6	*P. cyrtonema*	Shaowu, Fujian, China	29	*P. cyrtonema*	Loudi, Hunan, China
7	*P. cyrtonema*	Guangze, Fujian, China	30	*P. cyrtonema*	Yiyang, Hunan, China
8	*P. cyrtonema*	Guangze, Fujian, China	31	*P. cyrtonema*	Jiujiang, Jiangxi, China
9	*P. cyrtonema*	Guangze, Fujian, China	32	*P. cyrtonema*	Pingxiang, Jiangxi, China
10	*P. cyrtonema*	Guangze, Fujian, China	33	*P. cyrtonema*	Shaoxing, Zhejiang, China
11	*P. cyrtonema*	Guangze, Fujian, China	34	*P. cyrtonema*	Lishui, Zhejiang, China
12	*P. cyrtonema*	Lu'an, Anhui, China	35	*P. cyrtonema*	Qingyuan, Guangdong, China
13	*P. cyrtonema*	Guangze, Fujian, China	36	*P. filipes*	Guangze, Fujian, China
14	*P. cyrtonema*	Guangze, Fujian, China	37	*P. filipes*	Guangze, Fujian, China

15	*P. cyrtonema*	Guangze, Fujian, China	38	*P. filipes*	Lu'an, Anhui, China
16	*P. cyrtonema*	Loudi, Hunan, China	39	*P. filipes*	Guangze, Fujian, China
17	*P. cyrtonema*	Yichang, Hubei China	40	*P. filipes*	Sanming, Fujian, China
18	*P. cyrtonema*	Guangze, Fujian, China	41	*P. filipes*	Sanming, Fujian, China
19	*P. cyrtonema*	Guangze, Fujian, China	42	*P. kingianum* var. *grandifolium*	Chengdu, Sichuan, China
20	*P. cyrtonema*	Guangze, Fujian, China	43	*P. kingianum*	Wenshan, Yunnan, China
21	*P. cyrtonema*	Guangze, Fujian, China	44	*P. alternicirrhosum*	Ya'an, Sichuan, China
22	*P. cyrtonema*	Sanming, Fujian, China	45	*P. macropodum*	Tai'an Shandong, China
23	*P. cyrtonema*	Lu'an, Anhui, China	46	*P. odoratum*	Guilin, Guangxi,China
